# Education Research: Exploring the Impact of Standardized, Condition-Specific Note Templates on Quality Metrics and Efficiency in Multiple Resident Clinics

**DOI:** 10.1212/NE9.0000000000200200

**Published:** 2025-03-05

**Authors:** Andrew Breithaupt, Sonam Mohan, Robert Thombley, Samuel D. Pimentel, Vanja C. Douglas

**Affiliations:** 1 Department of Neurology, Emory University School of Medicine, Atlanta, GA;; 2 Department of Neurology, Kaiser Permanente, San Jose, CA;; 3 Division of Clinical Informatics and Digital Transformation, Department of Medicine, University of California, San Francisco, CA;; 4 Department of Statistics, University of California Berkeley, CA; and; 5 Department of Neurology, University of California, San Francisco, CA.

## Abstract

**Background and Objectives:**

Electronic health record documentation burden negatively affects physician satisfaction and patient care. Although well-constructed notes are important for care quality and safety, most note templates are created and maintained by individual physicians, leading to inefficiency and variable note quality. This study aimed to assess whether standardized, condition-specific note templates could enhance the efficiency and quality of notes written by neurology residents in the outpatient setting.

**Methods:**

In a quality improvement study with a randomized, nonblinded design from July 2021 to June 2022, neurology residents were assigned standardized templates for epilepsy, headache, and Parkinson disease (PD) in 2 outpatient clinics. The standardized templates were created with input from specialists in these disorders. Efficiency was gauged based on the time and characters involved in note writing while quality was assessed by adherence to American Academy of Neurology quality metrics for each condition through chart review. A qualitative survey gathered resident opinions on the templates. Linear regression models were used in the efficiency and quality analyses.

**Results:**

The study included 23 of 34 neurology residents. Templates were used in 36% of eligible encounters over the first 6 months of the study and 65% over the last 6 months. No significant difference in time spent on note writing was observed between the template and nontemplate groups. While both groups showed similar quality measures across most domains, the template group documented quality measures more consistently for driving status in epilepsy (92% vs 53%, *p* = 0.002), medication-related motor symptoms in PD (95% vs 50%, *p* = 0.01), and lifestyle changes in headache management (77% vs 21%, *p* = 0.005). Resident feedback suggested that the templates facilitated clinic workflows and prompted more thorough patient inquiry.

**Discussion:**

Standardized, condition-specific templates improved documentation of quality metrics without increasing time spent. Despite initial low uptake of template use, an increase was observed over time, indicating potential for wider acceptance with implementation efforts. These templates, updated and maintained by subject matter experts, serve as an opportunity to incorporate quality care checklists and knowledge into a clinician's workflow. This warrants further research into template implementation and its effects on care quality and education for neurologists and generalists.

## Introduction

There are multiple reports of physician dissatisfaction with electronic health records (EHRs), often citing clinical notes that are longer but not necessarily full of useful information.^1^ A National Physician Poll revealed that 74% of providers cite an increase in the number of hours worked each day with the use of an EHR, with a similar percentage citing subsequent burnout as a consequence.^2,3^ Residents and program directors identify documentation burden as a problem for trainees.^4^ In the outpatient setting, time spent on documentation increases cognitive load^2^ and takes away from other clinic responsibilities including spending time with patients during clinic visits, calling patients with results, prescribing medication refills, and managing EHR clinical messages. It also may contribute to resident duty hour violations. Despite the burden of note writing, well-written notes can greatly affect care quality and patient safety.^5,6^

Numerous studies have shown that note templates may reduce documentation burden and improve note quality. A progress note template introduced in the inpatient setting with associated training on using the template significantly reduced the length and time medicine interns spent on progress notes and improved note quality.^7^ Note templates led to minor improvements in documenting indications for imaging studies^8^ and improved operative notes.^9,10^ There have been mixed results on whether they improve documentation of physical examination findings.^11-13^

Applying cognitive load theory, a structured, well-organized template with expert-derived questions and management considerations for specific conditions may facilitate efficient navigation and documentation, thereby aiding learners by reducing extraneous cognitive load through the split-attention principle.^14,15^ This is in contrast to a note template created by an individual provider with varying levels of efficiency and quality. Standardized, condition-specific note templates in the outpatient setting could enable residents to finish their notes faster while ensuring that they have greater cognitive space to learn and avoid missing key items of the history, examination, or diagnostic workup. They could also enable better compliance with quality metrics. We hypothesized that implementing a set of standard note templates for epilepsy, headache, and Parkinson disease (PD) would increase resident efficiency and improve quality of care for patients with these conditions.

## Methods

We conducted a quality improvement (QI) study with a randomized, nonblinded design among neurology residents at the University of California San Francisco (UCSF) in their outpatient resident clinics at UCSF and Zuckerberg San Francisco General Hospital (ZSFG).

### Study Setting

At our institution, neurology residents create their own condition-specific templates or receive them from other residents. The result is several different templates for the same condition with varying levels of efficiency and quality, and no official approval or oversight beyond attending physician attestation. The neurology resident QI team decided to address this issue as part of an annual, hospital-wide, graduate medical education QI incentive program. This is a program available to all residency programs at UCSF, in which the neurology residency program participates every year with all residents participating and a resident QI team choosing a project. This program would provide a monetary incentive of $400 for all residents, regardless of study participation, at the end of the academic year if at least 70% of applicable PD, headache, and epilepsy encounters across all residents used the standardized note templates. This in-process metric was chosen as a target rather than documentation of quality metrics because it was unknown whether the template would indeed improve documentation of quality metrics. The target of 70% was chosen by leadership of this graduate medical education QI incentive program based on their experience using participation metrics in residency projects (including residency programs beyond neurology). Compliance for the QI incentive program was measured only for the templates residents chose (if not study participants) or randomly assigned (study participants); for example, if a resident chose or was assigned the headache and PD templates, their compliance would only be measured for encounters where the primary diagnosis was related to headache or PD and not encounters related to seizure. The neurology resident QI team involved 3 postgraduate year (PGY)-2 residents, 1 PGY-3 resident, and 4 PGY-4 residents with varying levels of involvement.

**Figure 1:**
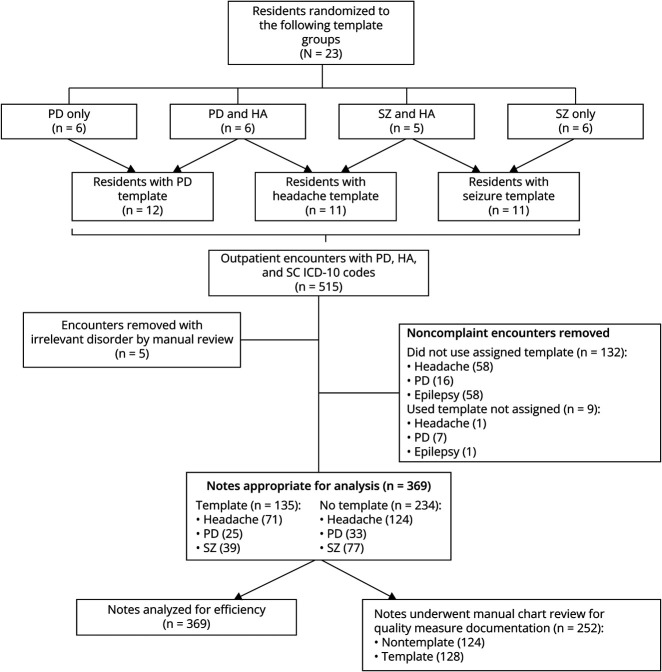
Resident Randomization and Note Selection for Analysis HA = headache; PD = Parkinson disease; SZ = epilepsy (seizure).

Neurology residents who chose to participate in this study (n = 23) were randomized to 1 of 4 template groups: PD template only (n = 6), PD and headache templates (n = 6), epilepsy template only (n = 6), or epilepsy and headache templates (n = 5). This ensured that each template was used by half of the participants while the other half served as controls. Because the EHR allows unregulated sharing of templates, we could not prevent residents from sharing templates to others outside their randomized group, so we only analyzed encounters in which a resident's note was concordant with their randomization group. For example, if someone who was randomized to the epilepsy template group used a headache template, that note was not analyzed (Figure 1). To limit bias, participating residents were asked not to use or review templates they were not assigned, and these residents were blinded to the specific quality metrics being assessed. Residents who chose not to participate (n = 11) in the randomized study were given access to all 3 templates, but they chose 2 for compliance tracking within the QI program. Documentation quality metrics were not collected for these residents.

Templates were developed in consultation with clinical faculty who were experts in the treatment of movement disorders, epilepsy, and headache and were distributed using the electronic medical record system. The American Academy of Neurology quality measures were consulted to ensure that they were included in the notes in addition to other elements our domain experts felt were clinically meaningful to help residents with diagnosis and management. Once the content of the notes was finalized, a clinical informaticist within the neurology department reviewed the templates to optimize usability.

### Identifying Applicable Notes Using ICD-10 Codes

The International Classification of Diseases, 10th Revision (ICD-10) codes were used to identify notes addressing PD, epilepsy, and headache. Several validation studies^16-20^ were used for each neurologic disorder to ensure that all applicable ICD-10 codes were included. In addition, we used the Agency for Healthcare Research and Quality Clinical Classifications Software Refined ICD-10-CM tool^21^ to identify any additional codes not mentioned in these articles to increase our sensitivity for identifying all relevant notes. Notes identified using these ICD-10 codes were reviewed to ensure that they were applicable to PD, epilepsy, or headache. Five notes captured using ICD-10 codes but found on chart review to be addressing a different diagnosis were excluded. All ICD-10 codes used can be found in the eAppendix.

### Measuring Efficiency

All notes written by participating residents and concordant with their randomization for patients with a primary ICD-10 diagnosis code for PD, epilepsy, and headache seen during the time frame of the study were evaluated. Efficiency was measured by time spent writing the visit note (including all edits performed during precharting, the visit itself, or after the visit) and the number of characters manually typed in the note.

### Measuring Complexity

Because the complexity of a visit can affect efficiency, we collected Current Procedural Terminology (CPT) Evaluation and Management (E/M) codes to determine the level of service for each visit. These codes were chosen by attending physicians at UCSF. CPT codes were not used for billing purposes at ZSFG, but the EHR system required residents to select a CPT code to close the patient encounter. This procedural nuance led to the inclusion of CPT codes for ZSFG encounters without attending oversight, despite the lack of formal training for residents on CPT code selection. Fifty-seven percent of encounters were from ZSFG while 43% were from UCSF.

### Choosing Quality Metrics

Based on American Academy of Neurology quality measure definitions and input from our specialists, we identified 4 measures of note quality for each of our 3 conditions believed to be clinically meaningful but often neglected. Of note, although driving is a retired measure, our epileptologists believed that this was important both clinically and from a medicolegal perspective because physicians in California are required to report patients with disorders characterized by lapses of consciousness to the Department of Public Health.^22^ The quality measures used were as follows:

1. Epilepsy^23^
  i) Screening for depression and anxiety was performed
  ii) Women of childbearing potential (guidelines defined as 12–44 years) with epilepsy were counseled regarding contraception (i.e., antiseizure medication interactions with oral contraceptives)
  iii) Women of childbearing potential with epilepsy were counseled regarding folate use, contraception, and teratogenesis
  iv) The patient's driving status was recorded, and if they should not be driving, appropriate measures were taken
2. Parkinson disease^24^
  i) Documentation of any falls was performed
  ii) Medication-related motor complications were discussed
  iii) Psychiatric disorders or disturbances were noted
  iv) Counseling about maintaining an exercise regimen was performed
3. Headache^25^
  i) Headache frequency was documented
  ii) Counseling about modifiable lifestyle and chronification factors related to headache was performed
  iii) Documentation of treatment offered for acute migraine attacks was performed
  iv) Documentation of treatment offered for preventive therapy was performed

We then reviewed resident notes to determine whether they included documented evidence that the note met the minimum standard for the quality measures, relevant to the condition addressed by the visit. All patient encounters using a template concordant with each resident's randomization were analyzed by the lead author A.B. for quality measures through a chart review, with exception of 7 encounters that were not closed at the time of review (n = 128). We identified a similarly sized set of control encounters (encounters without a template, n = 124) and used the following decision rule to choose which encounters we reviewed: within each disease category, a control was always chosen within 3 months of the encounter using the template (assuming that residents may improve with documenting quality measures over time regardless of the template group), and if there were multiple control notes within 3 months of 1 encounter using a template, we avoided analyzing notes of the same resident within that disease category more than once to ensure that as many residents as possible were represented. A 1:1 ratio of template to control encounters was chosen to balance feasibility of chart review with type 1 error. Each quality measure was coded as a 1 if it was documented as being addressed during that encounter; otherwise, it was coded as a 0. For some quality measures, such as those pertaining to women of childbearing potential with epilepsy, the total number of qualifying cases was too small to draw conclusions.

### Assessing Resident Opinions

Qualitative feedback was gathered through quarterly meetings with residents and through an anonymous electronic survey using Qualtrics (Qualtrics, Provo, UT).^26^ All residents, regardless of participation in the study, were surveyed as part of the QI program. The survey was distributed from July 2021 to July 2022. In the survey, Likert scales were used to measure opinions about the standardized note templates created for this study. Because all residents were randomized to at least 1 template or given access to all templates if they did not participate in the study, comparisons between residents regarding opinions measured by the survey were not made.

### Statistical Analysis

The primary analysis was per-protocol (PP) to evaluate the efficacy of standardized note templates when used as intended, which only included encounters where residents appropriately used the templates they were assigned (treatment group) or appropriately used their own templates (control group). While an intention-to-treat (ITT) analysis measures the real-world effectiveness of an intervention as assigned, our goal in this study was to evaluate the effectiveness of note templates when used as intended and not to evaluate the effectiveness of a rollout of note templates in a resident clinic. Adherence to template use was variable, anecdotally because of residents forgetting rather than systemic barriers. As a result, ITT analysis would dilute the intervention's effects and limit our ability to understand the templates' true impact. ITT was performed as a secondary analysis for efficiency data, with results in eTable 2.

Linear regression models were used to estimate study effects. Models incorporated an indicator for template usage, clinic site (UCSF or ZSFG), level of service billed, and month effects to control for any potential seasonal or time-related biases, such as residents improving over the course of the year or improvements made in the notes for efficiency.

Given that each resident documented multiple encounters, we applied a cluster-randomized design, treating each resident as a “cluster” to account for within-resident correlation. This design was chosen for 2 main reasons:

1. Addressing within-resident correlation: Outcomes from encounters documented by the same resident are likely correlated because of resident-specific factors, such as note-writing style, experience level (PGY), and familiarity with patients. Clustering by resident helped adjust for this within-resident dependency.
2. Aligning with the randomization structure: Because each resident intermittently used the assigned template to which they were randomized, clustering by resident enabled us to assess the impact of template usage within the consistent exposure context of each resident's assigned template group.

Randomization tests, which permuted observed treatment assignments while holding outcome values fixed, were used to assess statistical significance.^27,28^ One thousand random permutations were used in the clustered randomization test to ensure stable inferences. This method was used to compare time spent writing the visit note, the number of manually entered characters used in notes, level of service billed, and the proportion of notes satisfying each quality measure between template and nontemplate encounters. This statistical approach allowed us to evaluate and compare the impact of template usage on the efficiency and quality of patient encounters while adequately controlling for temporal variations and minimizing the need for strong assumptions about the distribution of study outcomes.

Outliers were identified in the time residents spent writing patient notes. This discrepancy likely arose from instances where residents left an active note-editing window open while performing other tasks in that patient's chart or the chart of another patient. In such cases, the EHR would continue to record this time as time spent writing a note. Two outliers were identified in the nontemplate headache group, with time spent in the patient's chart exceeding 800 minutes, and 2 outliers were identified in the nontemplate seizure group, with time spent in the patient's chart exceeding 1,500 minutes. To ensure the robustness of our analysis, we conducted our evaluations both with and without these outliers. All statistical analyses were completed in R version 4.3.2.

### Standard Protocol Approvals, Registrations, and Participant Consents

This research was approved by the Institutional Review Board of UCSF (protocol number 19-28438), and all participants provided informed consent.

### Data Availability

Anonymized data can be made available by request from any qualified investigator.

## Results

### Participant and Note Characteristics

All 34 neurology residents were included in the residency-wide QI project from July 2021 to June 2022. The evolution of the intervention and resident feedback are described further as part of this QI program. Twenty-three residents chose to participate in the study, with similar representation from each class: PGY-2 (38%), PGY-3 (33%), and PGY-4 (29%). The total number of notes included in the analysis is provided in Table 1. The compliance rate, efficiency, and quality metric results mentioned further are specific to the residents participating in this study.

**Table 1:** Note Characteristics

Condition	Total	No template, n (%)	Template, n (%)
Epilepsy	116	77 (66.4)	39 (33.6)
Headache	195	124 (63.6)	71 (36.4)
PD	58	33 (56.9)	25 (43.1)

Abbreviation: PD = Parkinson disease.

### Residency QI Project: Evolution of the Intervention and Process Measures

The intervention began with an educational session on the templates and their use, followed by quarterly meetings, emails, and surveys to gather resident feedback and make iterative improvements for user experience but content was not altered (e.g., changing fill-in-the-blank style paragraphs to bullet style checklists). The final templates can be found in the eAppendix. To improve template usage, a root cause analysis and fishbone gap analysis were completed at the end of the first quarter, which can be seen in eFigures 1 and 2. This led to the following interventions: starting in October 2021, (1) updates regarding template usage were given quarterly through email and didactic sessions, (2) monthly reminder emails were sent to residents who did not use the official templates during the previous month, and (3) reminder emails were sent to residents during their clinic weeks; starting in late November 2021, laminated reminders were placed on all outpatient clinic computers used by neurology residents; and starting in December 2021, “dotphrases” were introduced, allowing residents to incorporate the history of present illness (HPI) and assessment and plan (A&P) portions of the templates into their existing notes or preferred general templates.

### Study-Specific Compliance Rates

The compliance rate with the templates for the entire study period, measured by the percentage of applicable encounters where residents used their assigned template, was 50.5%. The compliance rate increased with each PGY (38%, 49%, and 60% for PGY-2, PGY-3, and PGY-4). Compliance overall increased throughout the study year, with an average compliance rate of 36% from July 2021 to December 2021, and after the interventions outlined above from late October 2021 to December 2021, an average rate of 65% from January 2022 to July 2022 was observed. The evolution of this compliance rate over time by month can be seen in eFigure 3. Noncompliance with using unassigned templates was 4%, with 9 of 243 encounters using a template note that was not assigned to the resident (Figure 1).

### Analysis of Efficiency

There was no statistically significant difference between the template and nontemplate groups with or without outliers in the time spent writing the note for each encounter, although notes documented for seizures used 827 more characters (*p* = 0.025). Including the outlier cases resulted in higher mean time spent in the chart of patients with headache and seizure in the nontemplate group, but the difference was not significant in the cluster randomization analysis. Table 2 summarizes the results of the PP analysis excluding outliers. Results with outliers included can be found in eTable 1. The results with an ITT analysis differed by showing no significant difference with characters typed in the seizure follow-up encounters (eTable 2).

**Table 2:** Time in Minutes Spent Writing Visit Notes and Manually Typed Characters in Notes

Note category	Writing visit note time (minutes)				Manually typed characters in notes			
	Mean (SD)		Cluster randomization analysis		Mean (SD)		Cluster randomization analysis	
	Template	Nontemplate	Estimated effect^a^	*p* Value	Template	Nontemplate	Estimated effect^a^	*p* Value
HA new	91.9 (78.9)	90.6 (37)	−2.4	0.875	2,865 (839)	3,329 (1,032)	−480	0.251
HA follow-up	89.7 (54.5)	83.4 (68.1)	9.6	0.649	2,155 (936)	2,552 (1,238)	−270	0.481
HA total	90.4 (62.5)	85.6 (60.4)	−2.1	0.896	2,375 (960)	2,790 (1,228)	−360	0.329
SZ new	112 (46.5)	86 (49.7)	9.8	0.827	3,393 (838)	3,795 (989)	−309	0.694
SZ follow-up	69.1 (39.3)	76.5 (46.7)	−1.1	0.940	2,735 (1,246)	2,005 (845)	827	0.025
SZ total	74.6 (42.2)	78.1 (47)	2.3	0.875	2,819 (1,214)	2,330 (1,110)	481	0.203
PD new	110.2 (44.4)	77.3 (42.9)	−32.0	1.000	4,507 (1,113)	3,995 (661)	−298	0.714
PD follow-up	79 (59.3)	96.5 (59.7)	−8.9	0.734	2,252 (915)	2,773 (1,065)	−646	0.087
PD total	85.2 (57.2)	94.7 (58.1)	−3.7	0.860	2,703 (1,310)	2,884 (1,087)	−94	0.813

Abbreviations: HA = headache; PD = Parkinson disease; SZ = epilepsy (seizure).

a Negative estimates signify less time or characters typed with the template group.

### Analysis of Quality Measures

**Figure 2:**
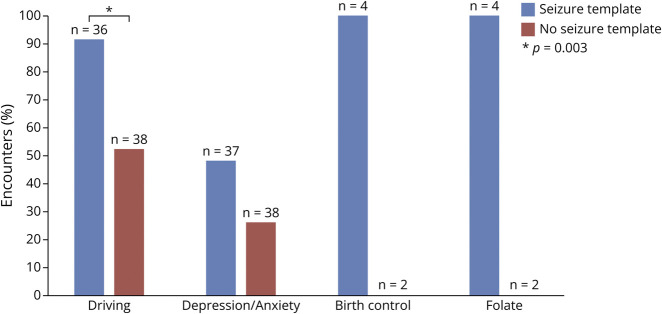
Quality Documentation in Epilepsy Encounters Approximately 50% of encounters documented asking about depression and anxiety in the template group compared with approximately 25% in the nontemplate group. Driving, birth control, and folate use quality metrics are discussed above.

**Figure 3:**
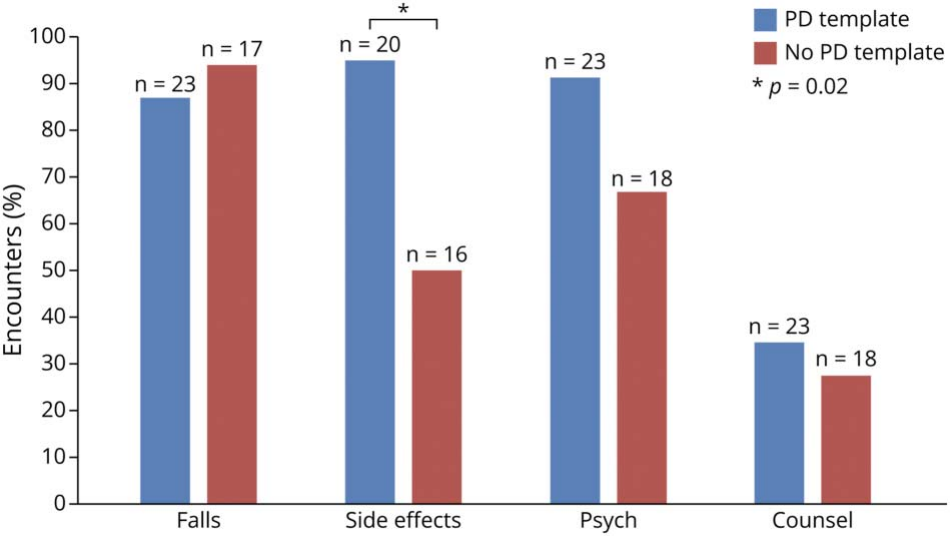
Quality Documentation in PD Encounters Asking about falls and counseling about exercise were documented in approximately 90% and 30% of encounters, respectively, in both the template and nontemplate group. Asking about psychiatric disturbances was documented in approximately 90% of the template group encounters vs approximately 65% of nontemplate group encounters. PD = Parkinson disease.

**Figure 4:**
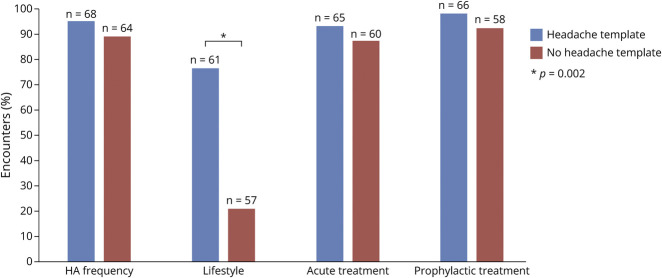
Quality Documentation in HA Encounters Asking about headache frequency and discussing acute and prophylactic treatment options were documented in approximately 90% of encounters in both the template and nontemplate group. HA = headache; Tx = treatment.

The template group was more likely to document the driving status of a patient with seizures (92% vs 53%, *p* = 0.003; Figure 2), ask about motor symptoms related to medications in patients with PD (95% vs 50%, *p* = 0.02; Figure 3), and discuss lifestyle changes for patients with headache (77% vs 21%, *p* = 0.002; Figure 4). There was a higher frequency of the template group documenting discussions of depression and anxiety in patients with seizures compared with the control group (48% vs 26%), but this difference was not statistically significant (*p* = 0.055). While the sample size was too small to analyze, all 4 applicable encounters in the template group asked about birth control and folate use in patients of childbearing potential with seizures while neither of the 2 applicable encounters in the nontemplate group addressed this topic.

### Analysis of Complexity

The complexity was significantly lower in the seizure follow-up template group (*p* = 0.02) in our primary PP analysis (eTable 4). There was no significant difference in the ITT analysis (eTable 5).

### Residency QI Project: Resident Feedback

During resident meetings, the most common critique raised was that utilizing the note template for follow-up visits precluded use of the copy forward function when the previous visit did not use the template. Nineteen residents responded to the survey (56%), which asked residents to answer survey questions comparing their experience using these standardized note templates with their usual method (using their own note template). Four of the residents surveyed did not use the new templates because they were comfortable with their own previous templates. Most of the 15 residents who used templates reported that templates made it easier to get through their clinic visit and complete their clinic note, prompted them to ask questions they may not have remembered otherwise, and included information that is helpful (Table 3). Further information about these responses, including responses by year of residency and the degree to which a resident agreed or disagreed, can be found in eTable 3.

**Table 3:** Summary of Resident Opinions Regarding Usefulness of the Templates

Prompts	Minimum^a^	Maximum^a^	Mean	SD	Count
I like using these templates	1	5	4.13	1.02	15
Completing the clinic note is easier	2	5	3.87	1.15	15
Navigating through the template is easy	2	5	4.00	0.97	15
Going through the clinic visit is easier	2	5	3.93	0.96	14
The note templates prompt me to ask questions I may not have remembered to ask otherwise	2	5	4.20	0.83	15
The information included in the templates is helpful	2	5	4.20	0.83	15
I find the templates too cumbersome or lengthy	1	5	2.21	1.15	14
The templates are causing me to spend more time on documentation	1	5	2.14	1.12	14

a Likert scale: 1 = strongly disagree, 2 = somewhat disagree, 3 = neither agree nor disagree, 4 = somewhat agree, 5 = strongly agree.

## Discussion

Standardized note templates designed for specific disorders can improve outpatient resident documentation adherence to quality metrics without increasing the amount of time spent on notes. Note templates also did not result in an increase in characters manually typed or result in lower level of service billed, with the exception of seizure follow-up notes. The randomized design is a strength of this study, which mitigates the selection bias of trainees who are enthusiastic about a specific template. Past studies looking at note templates among trainees have used before/after designs, which are difficult to interpret because trainee improvement over time through experience and learning makes it difficult to isolate the effect of templates on efficiency and quality. An additional strength of this study is the ability to include more accurate measures of efficiency by using the time spent writing the note and manual characters typed.

Despite no significant difference in time spent in the note, 1 standardized note template, seizure follow-up, resulted in more characters typed, yet a lower level of service billed. This is counterintuitive, although the level of service billed should be interpreted with caution because billing at ZSFG is not used but residents have to assign a billing code to close the encounter, without any training in billing. Although not statistically significant, new seizure and PD standardized note templates seemed to increase documentation times, possibly signaling greater comprehensiveness or residents feeling compelled to include all template elements for the QI project.

Past studies have had mixed results, but this study offers stronger support to those that have shown improved note quality.^7,8,10^ We anticipated that template adoption would be challenging because it required a change in workflow that was not guaranteed to increase efficiency or quality. The template design needed to balance efficiency, reducing resistance to adoption, with optimal quality. For example, normal physical examinations were prefilled to save time, although this risked documenting unperformed elements. Anecdotally, residents cited forgetting to use the templates as the main reason for low compliance, limiting the sample size. Residents attend outpatient clinic infrequently, and the templates applied to a small subset of cases, making habit formation difficult. Compliance rates for condition-specific templates vary in the literature, from 35% in residency programs to 97% among surgeons.^10-12^ However, template adoption and compliance increased over the course of our study with an overall plateau over the last few months both for residents participating in our study and those who did not, potentially because residents who adopted them found them helpful, although the inclusion of a financial incentive for template use regardless of the study participant and our interventions to increase template use likely also played a role. We note that, during the last month, compliance dropped to a level similar to the fourth month of the study, and we do not have further data to understand whether this was a trend that would have continued. Contrary to our expectations, compliance was lowest with the PGY-2 residents, despite these templates being introduced at the beginning of their training in neurology, and compliance increased with each year in training. While this initiative had champions evenly distributed across PGYs, a PGY-4 resident designed and predominantly led this study, which may have created a social proximity effect. Furthermore, for PGY-2 residents, there is so much to learn when starting out in clinic that incorporating new templates may have been lower on their list of priorities while more experienced residents may be more likely to have the bandwidth to incorporate a change in their workflow.

The study has several limitations. This study was isolated to 1 residency program, which may limit its generalizability to programs with different structures or EHR systems. However, several aspects of the study setting are likely shared across health care environments. The creation and use of note templates is common in clinic workflows, and the study spanned both a community-based, safety-net hospital and a tertiary academic medical center. The study also reflects common conditions faced by neurology residents who spend limited time in the outpatient setting. Real-time oversight was not feasible, and noncompliant encounters were identified only after monthly EHR data pulls. Therefore, there may be unmeasured reasons that led residents to choose to use templates for some encounters and not others, and that may have influenced efficiency metrics. The lack of real-time oversight may have contributed to low compliance, especially during the first few months of the study, leading to a low sample size. The chart review was not blinded, and thus, bias may have falsely elevated quality-of-care scores of the standardized note template group; however, we attempted to minimize bias by using quality metrics that could be scored with an objective yes/no grade (i.e., specific information was either included in the note or not). Furthermore, efficiency metrics were collected electronically and unbiased. Most of the E/M codes in our study were chosen by residents without formal training, making this study's proxy for visit complexity less reliable. Finally, the primary analyses were PP instead of ITT, which favors efficacy of the templates over real-world effectiveness of distribution of clinic templates.

While this study was performed in a resident outpatient clinic, the creation of standardized, shared note templates for specific disease entities has the potential to improve quality metrics without decreasing efficiency for other provider groups as well. The power of checklists to improve quality of care has been widely published for more than a decade now, but implementation of checklists can often be difficult, at least in part because of difficulty integrating them into established workflows.^29,30^ In this study, compliance increased for the more experienced providers, suggesting new note templates can be introduced and adopted by providers who have been using their own templates for at least 2 years with implementation efforts. Future research should focus on implementation efforts of these templates, which can include further iterations of the templates by identifying the highest yield quality metrics and consolidating essential content to increase adoption. Improved implementation of these templates could allow expanding investigation of standardized, condition-specific templates as a tool that general providers can use to manage many patients who cannot access a neurologist or other specialist.

## Glossary

CPT: Current Procedural Terminology
EHR: electronic health record
E/M: Evaluation and Management
ICD-10: International Classification of Diseases, 10th Revision
ITT: intention-to-treat
PD: Parkinson disease
PGY: postgraduate year
PP: per-protocol
QI: quality improvement
UCSF: University of California San Francisco
ZSFG: Zuckerberg San Francisco General Hospital
